# To the Surface and Back: Exo- and Endocytic Pathways in *Trypanosoma brucei*

**DOI:** 10.3389/fcell.2021.720521

**Published:** 2021-08-06

**Authors:** Fabian Link, Alyssa R. Borges, Nicola G. Jones, Markus Engstler

**Affiliations:** Department of Cell and Developmental Biology, Biocenter, University of Würzburg, Würzburg, Germany

**Keywords:** cell surface, African trypanosomes, endocytosis, exocytosis, membrane recycling, Rab, clathrin

## Abstract

*Trypanosoma brucei* is one of only a few unicellular pathogens that thrives extracellularly in the vertebrate host. Consequently, the cell surface plays a critical role in both immune recognition and immune evasion. The variant surface glycoprotein (VSG) coats the entire surface of the parasite and acts as a flexible shield to protect invariant proteins against immune recognition. Antigenic variation of the VSG coat is the major virulence mechanism of trypanosomes. In addition, incessant motility of the parasite contributes to its immune evasion, as the resulting fluid flow on the cell surface drags immunocomplexes toward the flagellar pocket, where they are internalized. The flagellar pocket is the sole site of endo- and exocytosis in this organism. After internalization, VSG is rapidly recycled back to the surface, whereas host antibodies are thought to be transported to the lysosome for degradation. For this essential step to work, effective machineries for both sorting and recycling of VSGs must have evolved in trypanosomes. Our understanding of the mechanisms behind VSG recycling and VSG secretion, is by far not complete. This review provides an overview of the trypanosome secretory and endosomal pathways. Longstanding questions are pinpointed that, with the advent of novel technologies, might be answered in the near future.

## Introduction

African trypanosomes rely on efficient strategies of immune evasion to successfully establish and maintain an infection in both their insect and mammalian hosts (Pays et al., 2014). In the bloodstream forms of *Trypanosoma brucei*, a dense coat of variant surface glycoprotein (VSG) constitutes the main virulence factor (Vickerman, 1969; Cross, 1975). The large repertoire of VSG genes coupled with periodic switches of the expressed VSG enables the parasites to change their surface epitopes, which allows an escape from the humoral immune response (Cross et al., 2014; Horn, 2014). VSG mRNA abundance varies slightly depending on the nature of the expressed VSG, but can amount to as much as ∼15% of total mRNA in *T. brucei* Lister 427 (Maudlin et al., 2021) and each trypanosome displays roughly 10^7^ VSG monomers on its cell surface (Jackson et al., 1985; Bartossek et al., 2017). Thus, assuming a cell cycle of approximately 6 h, this would require the production of roughly 28,000 VSG molecules per minute. In steady-state cells, around 90% of the entire VSG is displayed on the cell surface with the remaining 10% found in intracellular compartments (Grünfelder et al., 2002). Most of the intracellular VSG cargo is found in endosomes, which harbor 3-times as many VSG molecules than the biosynthetic organelles (Grünfelder et al., 2002).

**FIGURE 1:**
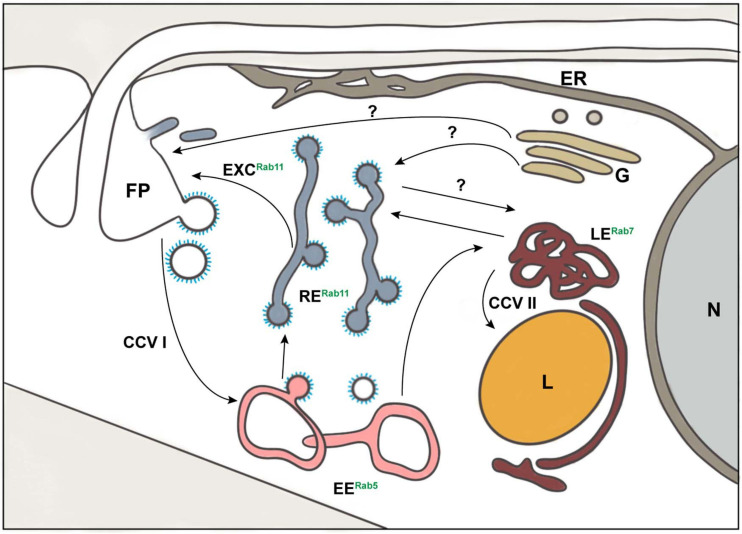
Schematic representation of exo- and endocytic pathways in *Trypanosoma brucei*. The known organelles involved in the processes of exo- and endocytosis in *T. brucei* are depicted: class I clathrin-coated vesicles (CCV I), class II clathrin-coated vesicles (CCV II), early endosomes (EE), endoplasmic reticulum (ER), exocytic carrier (EXC), flagellar pocket (FP), Golgi apparatus (G), lysosome (L), late endosomes (LE), nucleus (N), and recycling endosomes (RE). The endocytic compartment is marked by the presence of small GTPases of the Rab family: Rab5 (EE), Rab7 (LE), and Rab11 (RE). The arrows represent the direction of exo- and endocytic cargo of *T. brucei*. The question marks indicate pathways that may exist but are still unknown.

A second trypanosome immune evasion strategy is based on the high mobility of the VSG coat, which affords resistance to low levels of VSG binding antibodies. VSG molecules are covalently bound to a glycosylphosphatidylinositol (GPI) anchor which mediates their attachment to the outer leaflet of the lipid bilayer and facilitates lateral diffusion (Bülow et al., 1988; Hartel et al., 2016). Since the single flagellum is attached over the entire length of the trypanosome cell body and constantly beats, the directional motion generates a hydrodynamic flow on the cell surface, which is directed toward the posterior of the cell. The fluid flow specifically drags antibody bound VSGs to the flagellar pocket (FP) (Figure 1; Engstler et al., 2007). The process of antibody clearance is very fast (30–60 s), with the predicted clearance time varying as a function of the size of the antibody (Engstler et al., 2007). The parasite cell is shaped by a dense subpellicular microtubule cytoskeleton. This cortex is broken only at the FP rendering this invagination of the plasma membrane the exclusive place for endo- and exocytosis (Overath et al., 1997; Gull, 2003).

Both, maintenance of the VSG coat and the high rate of endocytic bulk membrane flow demand efficient intracellular transport machineries (Engstler et al., 2004, 2007; Manna et al., 2014). The components of the trypanosome secretory and recycling machineries localize to the posterior part of the cell, filling the volume between nucleus and FP (Grünfelder et al., 2003; Engstler et al., 2004; Field and Carrington, 2009; Manna et al., 2014). The exception to this is the endoplasmic reticulum (ER), which displays tube-like connections to the flagellar attachment zone (FAZ) and, therefore, longitudinally spans the entire length of the cell body (Vickerman, 1969; Lacomble et al., 2012). The fact that trypanosomes possess strictly localized exo- and endocytosis, combined with a high degree of cell polarization and full genetic tractability, makes them an attractive cellular model system. Furthermore, trypanosomes are placed in the group Discoba and hence, have diverged earlier than Opisthokonta, to which mammals and yeast belong (Burki et al., 2020). This opens avenues for modern comparative cell biology. Here, we will review the mechanisms of VSG sorting and recycling. For this purpose, we summarize the main aspects of the corresponding pathways in yeast and mammals first and subsequently highlight differences and gaps in our knowledge of the exo- and endocytosis machineries in *T. brucei*. In addition, we pinpoint how the advent of new and improved technologies might contribute to answering the remaining open questions.

## Biosynthesis and Sorting of Surface Molecules

The biosynthesis of molecules inside a cell involves many complex and highly regulated steps, which are collectively known as the biosynthetic pathway. Despite molecule specific differences, newly synthesized proteins follow a general route to their target compartments. During passage through the biosynthetic pathway, nascent proteins are modified and folded into their three-dimensional conformations.

### Entry Into the Endoplasmic Reticulum

Protein secretion starts with the targeted import of nascent polypeptides into the ER. In mammals and yeast, two main ways of protein import into the ER have been described: the co-translational and post-translational pathways (reviewed in Aviram and Schuldiner, 2017). In the co-translational pathway, the interaction between a signal peptide of the nascent protein with the signal recognition particle (SRP) targets the ribosome-nascent chain complex to the translocon (Akopian et al., 2013). In contrast, the post-translational translocation of proteins from the cytoplasm to the ER is SRP-independent. In this pathway, cytosolic chaperons bind to the nascent, signal peptide containing proteins and direct them to the translocon (Rapoport, 2007). The translocon is a conserved heterotrimeric membrane protein complex which transports the nascent proteins into the ER (Osborne et al., 2005). In *T. brucei*, both co- and post-translational pathways have been observed for the translocation of signal peptide containing proteins into the ER (Boothroyd et al., 1981; Lustig et al., 2007; Goldshmidt et al., 2008), suggesting conservation of the ER-import mechanisms. However, the post-translational pathway may be favored for GPI-anchored proteins (Goldshmidt et al., 2008) while co-translational translocation may be more important for polytopic membrane proteins (Boothroyd et al., 1981; Lustig et al., 2007).

### Processing and Quality Control in the ER

Once inside the ER lumen, the immature protein is exposed to ER-resident enzymes and undergoes several modifications assisted by chaperones and folding factors, which are usually referred to as the ER quality control system (Ellgaard and Helenius, 2003). A modification that occurs in nearly all glycoproteins, and represents a major function of the ER, is N-glycosylation (Helenius, 1994). Polypeptides bearing the glycosylation consensus sequence (N-X-S/T, where X can be any amino acid except for proline) serve as acceptors for a preassembled oligosaccharide (Welply et al., 1983; Mohanty et al., 2020). The reaction is catalyzed by oligosaccharyltransferase (OST) which transfers Glc_3_Man_9_GlcNAc_2_ *en bloc* to the asparagine side chain of the acceptor polypeptide (Mohanty et al., 2020). Processing of the glycan begins immediately after this transfer reaction: glucosidase I hydrolyzes the outermost glucose, which is followed by the sequential removal of the remaining two glucose residues through glucosidase II (Grinna and Robbins, 1979). Monoglucosylated oligosaccharides are recognized by the ER lectin-like chaperones calnexin and/or calreticulin (Williams, 2006; Rutkevich and Williams, 2011), which prevent aggregation, oligomerization and formation of non-native disulfide bonds (Moremen and Molinari, 2006). Trimming of the innermost glucose residue by glucosidase II releases the protein from calnexin/calreticulin. UDP-glucose/glycoprotein glucosyl transferase (UGGT) senses the folding state of released glycoproteins and, if the correct conformation has not been achieved, UGGT reglucosylates the N-glycan to enable another cycle of calnexin/calreticulin assisted protein folding (Solda et al., 2007; D’Alessio et al., 2010). Finally, correctly folded proteins are released from the cycle (Lederkremer, 2009). The remarkable ability of UGGT to bind to misfolded or incompletely folded glycoproteins was already reported in the early 1990s (Sousa et al., 1992), yet the complete recognition mechanism still remains unknown.

Proteins that are unable to acquire their native structure must be degraded to prevent accumulation of misfolded polypeptides in the ER. This degradation process is known as ER-associated degradation (ERAD), which usually involves recognition and retro-translocation from the ER to the cytosol, followed by ubiquitinoylation and proteasomal degradation (Brodsky, 2012). A special form of ERAD was reported for some misfolded GPI-anchored proteins in rat kidney cells (Satpute-Krishnan et al., 2014; Sikorska et al., 2016). Under acute ER stress, misfolded GPI-anchored proteins were exported from the ER, along the secretory pathway, to the plasma membrane from where they were eventually targeted to lysosomes for degradation (Satpute-Krishnan et al., 2014). This pathway was termed rapid ER stress-induced export (RESET) but may also operate constitutively in unstressed cells (Satpute-Krishnan et al., 2014).

Trypanosomatid protozoa are unable to synthesize dolichyl phosphate glucose (de la Canal and Parodi, 1987). Thus, while eukaryotic cells usually transfer the oligosaccharide Glc_3_Man_9_GlcNAc_2_ to their proteins, trypanosomatids attach unglucosylated glycans to the polypeptide chains (Parodi, 1993). In most eukaryotic organisms, the required OST consists of a multi-subunit protein complex with STT3A and STT3B as the catalytic domains. In contrast, *T. brucei* possesses three STT3 paralogs, with different acceptor specificities, but no other subunits of the OST complex (Izquierdo et al., 2009b). Thus, trypanosomes extended their glycosylation ability by duplication of the STT3 gene and diversification of STT3 specificity (Izquierdo et al., 2009b; Schwarz and Aebi, 2011). In addition, the recent finding of O-glycosylation of VSGs might indicate further unidentified biochemical diversity in protein processing factors in *T. brucei* (Pinger et al., 2018).

The orthologs of ER quality control proteins that were found and characterized in *T. brucei* include the reglucosylating UGGT, calreticulin, and glucosidase II (Conte et al., 2003; Jones et al., 2005; Izquierdo et al., 2009a). Although it is likely that the ER resident quality control machinery contributes to VSG folding, there is an ongoing discussion as to whether VSG folding is inherently energetically favorable and may therefore, not require chaperones (Rehaber et al., 1990). While some evidence suggests that VSGs might be synthesized in 2–3-fold excess, with their relative abundance regulated by an active ERAD (Field et al., 2010; Manna et al., 2014), other studies found no evidence for rapid degradation and promote a model whereby VSG synthesis is precisely regulated (Tiengwe et al., 2016). Interestingly, recent findings are adding a new piece to this puzzle. Aroko et al. (2021, preprint) demonstrated that targeting of several abundantly expressed proteins to the ER leads to a downregulation of mRNA levels of the endogenous VSG. Thus, they suggested that feedback generated at the ER has a central role in regulating VSG mRNA amounts.

### Export From the ER

Proteins exit the ER at specific locations named ER exit sites (ERES) via vesicles coated with coat protein (COP) II. The highly conserved and essential COP II coatomer is generally composed of two protein complexes Sec23/Sec24 and Sec13/Sec31, and the small GTPase Sar1 (Lopez et al., 2019). Vesicle formation is initiated by Sec12, a guanine exchange factor localized in the ER, which activates Sar1 (Nakano and Muramatsu, 1989; Barlowe and Schekman, 1993; Futai et al., 2004). Activated Sar1 embeds into the ER membrane and recruits the first heterodimer Sec23/24 via a specific interaction with Sec23 to the ERES (Bi et al., 2002; Fath et al., 2007). This “prebudding complex” selectively binds secretory cargo mainly via interaction with Sec24 and to a lesser extent with Sec23 (Miller et al., 2003, 2005; Cai et al., 2007; Farhan et al., 2007). Following this, the heterotetramer Sec13/Sec31 is recruited, which stimulates membrane deformation and vesicle fission from the ERES (Matsuoka et al., 1998). While transmembrane secretory cargo is selectively recruited via direct interactions between their cytosolic motifs and Sec24 (Aridor et al., 1998; Kuehn et al., 1998; Miller et al., 2003), soluble and GPI-anchored proteins cannot interact directly with the COP II machinery (Barlowe, 2003). These proteins require transmembrane cargo receptors, such as p24, which link them to the COP II coat (Schimmoller et al., 1995). The subsequent transport of COP II vesicles from the ER to the Golgi is likely to be mediated by the small GTPases Rab1 and Rab2 (Tisdale et al., 1992).

In *T. brucei*, two isoforms each of Sec23 and Sec24 have been found, with TbSec23.2 and TbSec24.1 responsible for VSG export (Sevova and Bangs, 2009). While the abundance of synthesized and transported VSG may explain the requirement for an additional specific heterodimer, the mechanism by which VSGs are selectively incorporated into TbSec23.2/TbSec24.1 COP II vesicles remains unknown. Recently, it was hypothesized that p24 orthologs in *T. brucei* may facilitate VSG incorporation into COP II vesicles (Kruzel et al., 2017). Studies with TbRab1 and TbRab2 have validated the role of these GTPases in ER to Golgi and *intra*-Golgi transport (Dhir et al., 2004).

### Golgi Traffic

The Golgi apparatus is a central membrane organelle that has a function in glycan maturation, trafficking and sorting. Each Golgi stack is formed by several tightly aligned flattened cisternae, which are referred to as *cis*-, medial- or *trans*-compartments. While the *cis*-Golgi network receives cargo from the ER, the medial-Golgi cisternae contain glycosylation enzymes and process cargo proteins and lipids, and the *trans*-Golgi network (TGN) sorts cargo molecules for delivery to different destinations (e.g., to the cell surface or to vacuolar or lysosomal compartments) (reviewed in Di Martino et al., 2019; Huang and Wang, 2017). In addition, the TGN can also be separated from the Golgi stack and may act as an early endosome in yeast and plants, suggesting that this compartment might be an independent organelle that is distinct from the Golgi apparatus (Uemura et al., 2004; Dettmer et al., 2006; Staehelin and Kang, 2008; Day et al., 2018).

Surprisingly, the mechanism of transport through the Golgi, is still controversial and different models that are not mutually exclusive have been suggested. The most prominent models suggest stable cisternae with COP I vesicles transporting cargo between them or cisternal maturation with progressive movement of cisternae toward the *trans* face (reviewed in Glick and Luini, 2011). COP I vesicles are also responsible for Golgi retrograde transport which returns ER resident proteins to the ER lumen (Maier et al., 2001). However, the exact mechanism of delivery and the extent to which this occurs both remain unclear.

In *T. brucei*, the stable cisternae model offers the most likely explanation for *intra*-Golgi transport (Warren, 2013). TbRab18 (Jeffries et al., 2002), and TbRabX2 (also called TbRab31) (Field et al., 2000) have been associated with the Golgi apparatus and may be related to Golgi dependent transport pathways. In TrypTag.org, a project that aims to determine the localization of every trypanosome protein within the cell (Dean et al., 2017), TbRab6 (Tb927.2.2130) was also found to be located in the region of the Golgi.

### Post-Golgi Transport to the Plasma Membrane

Cargo proteins destined for the plasma membrane are loaded into post-Golgi carriers and follow the secretory pathway (Luini et al., 2005). In *Saccharomyces cerevisiae*, the molecules involved in this transport were identified by the isolation of *sec* mutants that were unable to secrete the extracellular enzyme invertase (Novick et al., 1980, 1981). Subsequent studies in yeast have strongly indicated an additional role of the Rab11 GTPase family in Golgi exit and transport to the sites of exocytosis (Jedd et al., 1997; Morozova et al., 2006; Lipatova et al., 2008). Secretory carriers are directed by tropomyosin-actin cables and delivered to the plasma membrane to which they are tethered by the exocyst complex prior to SNARE complex dependent fusion in a Sec4 GTPase manner (TerBush et al., 1996; Guo et al., 1999).

In *T. brucei*, the mechanism of transport from the Golgi to the FP remains elusive and it may be possible that different carriers, perhaps containing different cargos, are involved (Figure 1). The role and involvement of TbRab11 in the secretory pathway is still contentious. VSG has been demonstrated to be transported inside TbRab11-enriched exocytic carriers during VSG recycling using immunoelectron microscopy (Grünfelder et al., 2003). However, RNAi silencing of TbRab11 showed no effect on exocytosis of newly synthesized VSG in pulse-chase radiolabel experiments (Hall et al., 2005). In accordance with these results, another study, also using pulse-chase radiolabeling of VSGs, validated that depletion of TbRab11 had no impact on the transport of newly synthesized VSG (Umaer et al., 2018). However, no reports of TbRab11 negative secretory carriers can be found in the literature. This could be explained by the existence of a yet unidentified route from the Golgi to the FP that could easily have been overlooked due to the comparatively small proportion of biosynthetic VSG in the total intracellular VSG pool. Furthermore, the exocyst has been shown to be an important mediator of the late steps of exocytosis. This complex presents one subunit (Sec15) which interacts with TbRab11 (Boehm et al., 2017), highlighting once more the importance of this GTPase in the exocytosis process of *T. brucei*. Altogether, these findings suggest the need to characterize the role of TbRab11 in a more detailed manner. Is TbRab11 involved in post-Golgi transport to the FP? Is this transport mediated via endosomes? Does TbRab11 distinguish between VSG and other cargo transport? Another intriguing aspect regarding Golgi transport to the FP is related to the role of the cytoskeleton. The export of newly biosynthesized VSG from the Golgi has been reported to be independent of actin (Nolan and Garcia-Salcedo, 2008). No further reports about the involvement of cytoskeleton components in cargo transport exist, but it is unlikely that these pathways can be entirely independent of interactions with the cytoskeleton. Thus, the role of the cytoskeleton remains largely undefined.

## Endocytosis and Membrane Recycling

Endocytosis allows eukaryotic cells to internalize plasma membrane proteins and extracellular molecules for various physiological processes, including nutrient uptake, degradation, cell signaling and recycling. The endocytosis processes are generally divided into fluid-phase and receptor-mediated endocytosis. The first refers to the non-specific uptake of extracellular material, and the second is characterized by the triggered uptake of a ligand after binding to its receptor. Different endocytic pathways exist in eukaryotes, such as caveolae-mediated (Kiss and Botos, 2009) and raft-dependent endocytosis (Lajoie and Nabi, 2007). However, the major endocytic pathway is mediated by clathrin (Bitsikas et al., 2014), which explains why the term endocytosis is often used synonymously with clathrin-mediated endocytosis. In *T. brucei*, all endocytic processes, for both fluid phase and receptor-mediated uptake, are clathrin-mediated (Allen et al., 2003; Grünfelder et al., 2003; Engstler et al., 2004).

### Clathrin-Mediated Endocytosis and Endocytic Trafficking in Mammals and Yeast

Clathrin molecules are trimers composed of three heavy chains, each associated with one light chain, connected through a central core (Crowther and Pearse, 1981; Ungewickell and Branton, 1981). Due to the architecture of the molecule, clathrin trimers are called triskelions (Ungewickell and Branton, 1981). Clathrin triskelions can connect with one another to form flat lattices or a cage-like structure (Crowther and Pearse, 1981; Kirchhausen and Harrison, 1981; Sochacki and Taraska, 2019). The curvature of clathrin lattices linked to the membrane contributes to the forces required for local deformation, that culminates in pit formation and vesicle budding (Kirchhausen and Harrison, 1981; Kaksonen and Roux, 2018; Sochacki and Taraska, 2019). Flat hexagonal clathrin lattices need to reorganize to form the curved mix of pentagonal and hexagonal lattices (Sochacki and Taraska, 2019). The organization of clathrin triskelions into flat lattices has also been shown to occur spontaneously *in vitro* (Dannhauser et al., 2015). However, the conformational change from flat to curved clathrin lattices *in vivo* most likely demands energy and requires the participation of accessory molecules (reviewed in Kaksonen and Roux, 2018; Sochacki and Taraska, 2019). The recruitment of clathrin to the plasma membrane requires several proteins that form the pioneer module, a complex with several characterized components, such as F-BAR domain only protein 1 and 2 complex (FCHO1/2), adaptor protein 2 (AP-2), and epidermal growth factor receptor substrate 15 (EPS15) (Shih et al., 1995; Ma et al., 2016; Kaksonen and Roux, 2018). In addition, several molecules such as actin-related protein 2/3 complex (Arp2/3), protein-rich protein Las17 (Las17), type I myosins (Myo3/5), Sla2 and epsin 1 (Ent1) (Galletta et al., 2008; Cheng et al., 2012; Feliciano and Di Pietro, 2012; Kaksonen and Roux, 2018; Lizarrondo et al., 2021), contribute to polymerization of the actin cytoskeleton. Finally, the progressive invagination of the membrane is associated with a constriction process, which culminates in membrane scission (Kaksonen and Roux, 2018). Constriction involves actin, dynamin, and PI(4,5)P_2_ (reviewed in Mettlen et al., 2009). This phosphoinositide can bind to the epsin N-terminal homology (ENTH) and AP180 N-terminal homology (ANTH) domains of proteins and is considered critical for the recruitment of adaptors and formation of clathrin coated pits (CCP) (Wendland et al., 1999; Zoncu et al., 2007; Kaksonen and Roux, 2018), which bud as clathrin coated vesicles (CCVs). These CCVs are then uncoated in a process involving dephosphorylation of PI(4,5)P_2_ by inositol 5-phosphatases and disruption of the clathrin–clathrin interactions by HSC70 (Prasad et al., 1993; Ungewickell et al., 1995; Kaksonen and Roux, 2018). The uncoated vesicles are able to fuse with endosomes, a polymorphic, dynamic endomembrane system, commonly divided into subpopulations named early (or sorting) endosomes (EEs), late endosomes (LEs), and recycling endosomes (REs) (Goldenring, 2015; Naslavsky and Caplan, 2018). These different subclasses can be distinguished according to marker proteins enriched in their membranes, especially those belonging to the Rab family, as well as by differences in luminal pH (Feng et al., 1995; Rink et al., 2005; Wandinger-Ness and Zerial, 2014; Raiborg et al., 2015; Naslavsky and Caplan, 2018) which is controlled by a V-ATPase pump (Hurtado-Lorenzo et al., 2006; Scott et al., 2014).

Endocytosed cargo enters EEs first, from where the molecules are sorted for different fates, either degradation or recycling. When destined for degradation, the cargo passes through LEs before arriving in the lysosome. In the recycling pathway, there are two possible routes: anterograde transport, which returns the molecules to the cell surface, and retrograde transport, which transports cargo to the TGN (Cullen and Steinberg, 2018; Naslavsky and Caplan, 2018). The dynamics of cargo transport is still a topic of debate and distinct models have been proposed. The vesicular-transport model suggests that vesicles budding from steady-state organelles move their cargo to the different endocytic compartments. However, studies on living mammalian cells have suggested the existence of a maturation process in which these organelles/compartments are formed from progressive differentiation of endocytic vesicles (Rink et al., 2005; Scott et al., 2014; Langemeyer et al., 2018; Trivedi et al., 2020).

In mammals, the EEs have been characterized to be a vacuolar organelle associated with tubular membranous extensions (Cullen and Steinberg, 2018). They are further characterized by the presence of Rab4, Rab5, Rab10, Rab14, Rab21, and Rab22, which are found in distinct microdomains of the EE membrane (Scott et al., 2014; Naslavsky and Caplan, 2018). Inside the EEs, molecules destined for degradation are sorted with the participation of the endosomal sorting complex required for transport (ESCRT) into luminal invaginations of the EE membrane. These invaginations pinch off into the EE luminal space, thereby forming intralumenal vesicles (ILVs). The ILVs accumulate in vacuolar regions of EEs, which will detach and become free multivesicular bodies (MVBs) or endosomal carrier vesicles (ECVs). The MVBs/ECVs are subsequently transported on microtubules to a different location in the cell (Scott et al., 2014; Raiborg et al., 2015). Interestingly, live-cell microscopy studies suggested the maturation of these Rab5-positive MVBs/EVCs into Rab7-positive LEs, with Rab5 gradually replaced by Rab7 (Rink et al., 2005; Scott et al., 2014; Langemeyer et al., 2018). Thus, the LEs are mature MVBs with a different set of marker proteins and a more acidic pH. The morphology of LEs, with tubular-cisternae and multivesicular regions, is similar to that of EEs, which are also formed through ESCRT sorting into ILVs (Scott et al., 2014; Cullen and Steinberg, 2018). From the LEs, the cargo can follow either the degradation or the recycling pathway. For degradation, LEs fuse with lysosomes, forming a hybrid organelle referred to as the endo-lysosome. This transient organelle matures into the classical lysosome, which can be distinguished from LEs through its spherical and electron-dense structure (Rink et al., 2005; Scott et al., 2014). Cargo from the LEs that is not destined for the lysosome, can be routed to the cell surface through tubular-vesicular carriers which fuse with the plasma membrane (van Weering et al., 2012; Cullen and Steinberg, 2018). Another path back to the cell surface is via REs, which also have contact sites with EEs. This compartment is composed of highly dynamic tubular structures that are characterized by the presence of Rab11 and Rab8 (reviewed in Hsu and Prekeris, 2010; Scott et al., 2014; Goldenring, 2015). Interestingly, in polarized cells, Rab11 seems to participate in direct recycling of cargo to the plasma membrane while Rab8 functions in transport via the TGN (Hsu and Prekeris, 2010; Cullen and Steinberg, 2018). Rab11-positive vesicles that bud from REs are directed to the plasma membrane through interaction with actin-motor proteins (Ji et al., 2019). Thus, actin is not only involved in CCV budding but also in the transport of endocytic cargo. The retrograde transport of molecules to the TGN is mediated by the retromer complex. This complex is formed by at least three cargo selection molecules (Vps35, Vps26 and Vps29) that interact with members of the sorting nexin (SNX) family (reviewed in Scott et al., 2014).

### Endocytosis in *T. brucei*

**FIGURE 2:**
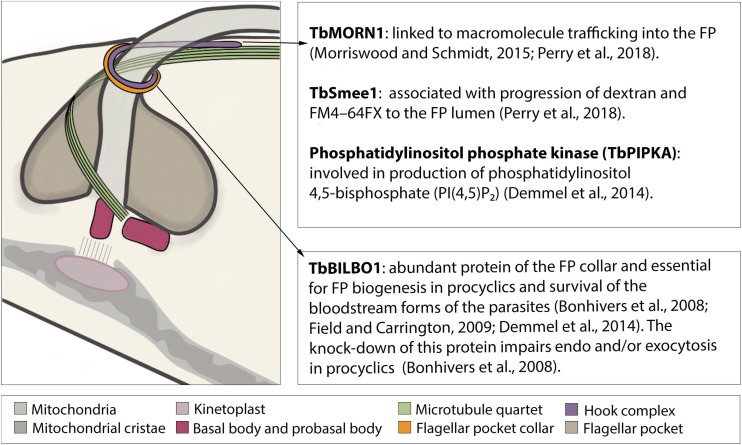
Schematic structure of the flagellar pocket with emphasis on its cytoskeleton components involved in endocytosis. The flagellar pocket (FP) of *Trypanosoma brucei* is a subdomain of the plasma membrane, corresponding to approximately 2–5% of its total area (Grünfelder et al., 2002; Engstler et al., 2004; Field and Carrington, 2009). On its most external face lies the flagellar pocket collar (FP collar) of which TbBILBO1 is the main component (Sherwin and Gull, 1989; Bonhivers et al., 2008; Lacomble et al., 2009). On top of the FP collar lies the hook complex (HC), a multiprotein structure, of which three components (TbMORN1, TbSmee1, and TbPIPKA) have been shown to be involved in endocytosis (Morriswood et al., 2009; Zhou et al., 2010; Esson et al., 2012; Demmel et al., 2014; Morriswood and Schmidt, 2015; Perry et al., 2018; Sajko et al., 2020).

*T. brucei* restricts both endo- and exocytosis to the FP (Figure 2; Vickerman, 1969; Overath et al., 1997; Gull, 2003; Engstler et al., 2004; Field and Carrington, 2009). Uptake from the FP involves the formation of CCPs (Morgan et al., 2001; Allen et al., 2003; Grünfelder et al., 2003; Engstler et al., 2004), which rapidly pinch off as CCVs with a diameter of 135 nm, known as class I CCV (CCV I) (Grünfelder et al., 2003; Overath and Engstler, 2004).

Clathrin can be recruited to the entire FP membrane with the exception of the region of the microtubule quartet (4MT) (Gadelha et al., 2009). This recruitment involves many proteins, which will interact with each other as well as with clathrin, cargo, other adaptors, and phosphoinositides. TbEpsinR and TbCALM are two characterized proteins that possess a phosphoinositide binding domain (ENTH and ANTH, respectively) and are critical for the recruitment of adaptors as well as for formation of CCPs (Gabernet-Castello et al., 2009; Manna et al., 2015). While TbEpsinR has been shown to colocalize with clathrin (Gabernet-Castello et al., 2009), the colocalization of clathrin and TbCALM has been inferred from their individual localization to the cytoplasmic side of the FP (Manna et al., 2015). Interestingly, TbEpsinR was distributed throughout the cytoplasm in clathrin-depleted cells leading to the proposition of targeting-dependency between TbEpsinR and clathrin (Gabernet-Castello et al., 2009). The depletion of either TbEpsinR or TbCALM led to a small reduction in receptor-mediated endocytosis and morphological aberrations in both CCP and CCVI (Manna et al., 2015). The simultaneous knockdown of both proteins led to FP enlargement and inhibition of endocytosis, suggesting redundancy in the roles of the two proteins (Manna et al., 2015).

The ATPase TbHsc70 co-precipitates with clathrin and colocalizes with clathrin-enriched structures (Adung’a et al., 2013). Knockdown of TbHsc70 reduces FITC-concanavalin A (ConA) trafficking into late endosomal compartments (Adung’a et al., 2013), suggesting a probable involvement in uncoating as observed *in vitro* and *in vivo* for mammals and yeast (DeLuca-Flaherty et al., 1990; Rapoport et al., 2008; Yim et al., 2010). Currently, no other proteins involved in clathrin uncoating have been detected in *T. brucei*.

Intriguingly, AP-2, one of the major clathrin recruiters in opisthokonts, has not been identified in the genome of African trypanosomes although it is present in the genome of other trypanosomes, such as *Trypanosoma cruzi*, *Trypanosoma theileri*, *Trypanosoma grayi*, and *Trypanosoma carassii* (Morgan et al., 2002; Manna et al., 2013). The authors suggested that the high density of the VSG coat would not require cargo selection/accumulation to be coordinated by AP-2. They also proposed that the absence of this protein could increase the speed of endocytosis by reducing the need for dephosphorylation reactions in the clathrin uncoating processes (Manna et al., 2013). However, clathrin uncoating is essential for cargo progression to the endosomal system. Therefore, dephosphorylation reactions are necessary in the presence of any clathrin-coat accessory molecules that bind to phosphoinositide, such as PI(4,5)P_2_ that is present in the FP membrane of *T. brucei* (Demmel et al., 2014). Thus, the abolishment of AP-2 dephosphorylation alone might not explain a faster endocytosis rate.

A proteomic approach revealed the existence of *T. brucei* clathrin-associating proteins (TbCAP) that had not been identified through *in silico* screening (Adung’a et al., 2013). Of these, eight were found to be exclusive to trypanosomatids: TbCAP116, TbCAP118, TbCAP125, TbCAP161, TbCAP186, TbCAP292, TbCAP334, and TbTOR-like 1 (Adung’a et al., 2013). Interestingly, knockdown of TbCAP100, TbCAP116, TbCAP161 and TbCAP334 decreased endocytosis of FITC-ConA at early time-points and led to FP enlargement, suggesting their involvement in clathrin assembly and VSG trafficking (Adung’a et al., 2013).

The cytoskeleton is another important factor in endocytosis. Recently, genetic screening confirmed the presence of actin and actin-related proteins in *T. brucei* (reviewed in Gupta et al., 2020). In bloodstream forms, actin is localized in the posterior region of the cell body, between the nucleus and kinetoplast, and its depletion causes impairment of endocytosis and enlargement of the FP (Garcia-Salcedo et al., 2004). The actin cytoskeleton also seems to play a role in the polarization of the mechanoenzyme TbMyo1 in the posterior region of bloodstream form cells (Spitznagel et al., 2010). Immunofluorescence assays on fixed cells revealed differences in both the number and the fluorescence intensity of TbMyo1 spots inside the population suggesting a dynamic nature of the protein (Spitznagel et al., 2010). Knockdown of TbMyo1 led to the impairment of endocytosis and changes in the subcellular distribution of clathrin, which clearly demonstrates its role in endocytosis (Spitznagel et al., 2010). Previous studies had suggested that dynamin, involved in CCV budding in mammals and yeast, was not involved in endocytosis in *T. brucei* (Morgan et al., 2004). However, the single dynamin-like protein of *T. brucei* has two paralogs (TbDLP1 and TbDLP2), which were found to have distinct expression patterns and functions depending on the life cycle stage of the parasite (Benz et al., 2017).

Compared to mammals and yeast, both the description and characterization of clathrin endocytosis in *T. brucei* lack details and require further analysis to be better understood. Among the questions to be elucidated are, for example, how clathrin recruitment and association with the CCP is coordinated and how cytoskeleton elements participate in vesicle budding from the FP membrane and in their subsequent transport. The use of proteomic methods to investigate the cohort of molecules involved in clathrin assembly in trypanosomes (Adung’a et al., 2013) highlighted the low conservation of the components compared to other eukaryote supergroups. This can either be explained by a reduced clathrin system in *T. brucei*, as suggested previously, or it demonstrates the need for new experimental strategies to complement *in silico* homology searches.

### Endosomal Trafficking in *T. brucei*

**FIGURE 3:**
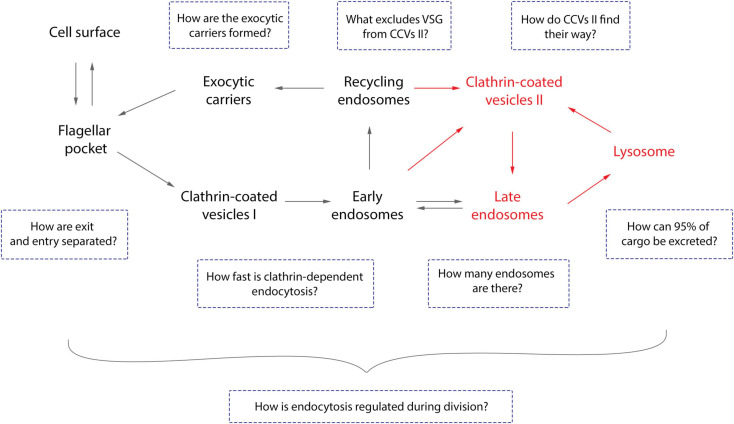
Open questions about endocytosis of trypanosomes. The general route of endocytosis and recycling in *Trypanosoma brucei* is depicted. The gray arrows represent the direction of all cargo while the red arrows refer solely to fluid-phase cargo. Endo- and exocytosis is restricted to a single site in trypanosomes: the flagellar pocket. Thus, the first intriguing question is how does the cell discriminate between cargo entering and leaving the pocket? All molecules are endocytosed via clathrin mediated endocytosis (CME), the recycling rates are incredibly fast, and *T. brucei* lacks AP-2 (Grünfelder et al., 2002; Morgan et al., 2002; Engstler et al., 2004). Therefore, the naturally following questions are: how fast is CME and does the lack of AP-2 influence this process? VSGs have never been observed in the lysosome and the route to this organelle is likely to be mediated by class II clathrin-coated vesicles (CCV II). So, the remaining questions here are how VSG is excluded from CCV II sorting and how these vesicles are routed to the lysosome. Furthermore, the number of endosomes in *T. brucei* cells and the origin of exocytic carriers remain elusive as does the mechanism of excretion of 95% of the cargo. Overall, the essentiality of the VSG coat for trypanosomes allied to the natural increase of volume of the cell during the cell cycle advocates for a precise regulatory mechanism to maintain the dense surface coat. However, such a mechanism has not yet been elucidated.

In *T. brucei*, all endosomal compartments are in the posterior part of the cell (Figure 1). All endocytosed cargo, VSG and fluid phase, pass through EEs (Grünfelder et al., 2003; Engstler et al., 2004). Following uptake, VSG starts to colocalize with EEs after 2.2 s and finally fills up to 85% of the compartment volume (Engstler et al., 2004). From this compartment, 53% of the VSG pool moves to the REs and returns to the surface, a route known as the fast route, which is completed within approximately 10 s (Engstler et al., 2004). Alternatively, 47% of VSGs are recycled through the slow route, which takes approximately 50 s to complete (Engstler et al., 2004). Here, VSG passes first from the EE to the LE, a path also followed by the fluid phase cargo, and then returns to the surface via the RE. Interestingly, while the fluid phase cargo moves from the LE to the lysosome for degradation no VSG was detected inside this organelle (Engstler et al., 2004). The retrograde transport of cargo to the TGN has not yet been characterized in *T. brucei*. Studies on the endosomal compartment of *T. brucei* have only been conducted on fixed samples. This, allied to the transient nature of the maturation model, limits our comprehension of the endosomal compartment in this organism. Nevertheless, the endocytic compartment of *T. brucei* also has the characteristic tubular-vesicular shape found in other organisms (Engstler et al., 2004). One molecule, TbMBAP1, was found in all endosomal membranes (Engstler et al., 2005). Four EE markers in this parasite have been identified through homology searches: one homolog of Rab4 (TbRab4), two homologs of Rab5, TbRab5A, and TbRab5B (Field and Boothroyd, 1995; Field et al., 1998), and one homolog of Rab21, TbRab21 (Ali et al., 2014). Studies using epifluorescence microscopy have suggested TbRab4 and both subpopulations of TbRab5 to be present in vesicles in the cytoplasm of bloodstream forms (Field et al., 1998). These vesicles were localized between nucleus and kinetoplast, which is where the endosomal compartment of *T. brucei* is found, and some overlap was observed (Field et al., 1998). However, it is crucial to remember that the resolution of widefield microscopy combined with the close proximity of different endocytic compartments does not allow an accurate localization. In addition, it is tempting to ask if these markers might all be part of a single structure with distinct subdomains. If that were the case, how is the cargo sorted inside this structure? To answer these questions (Figure 3), it is crucial to use super-resolution microscopy.

In bloodstream forms TbRab4 acts in fluid phase transport to the lysosome and in the accumulation of the constitutive transmembrane glycoprotein p67 (Hall B. S. et al., 2004; Peck et al., 2008). TbRab5A was related to IgG, transferrin, VSG, and invariant surface glycoprotein (ISG) 65 transport, while TbRab5B has so far only been linked to ISG100 (Field et al., 1998; Chung et al., 2004; Hall B. et al., 2004).

The expression level of TbRab21 is low in the bloodstream forms of the parasite, but the overexpression of tagged TbRab21 revealed its localization in the posterior part of the cell (Ali et al., 2014). Immunofluorescence assays showed a partial overlap of TbRab21 with clathrin, TbRab5A and TbRab11, and juxtaposition with p67. However, some variation in the relative position of TbRab21 to TbRab11 and p67-positive structures was observed within the population (Ali et al., 2014). These variations in position could again be related to the limited resolution of the microscopy techniques used in the study along with cell-to-cell expression level variations. Furthermore, despite partial colocalization with TbRab5A, knockdown of TbRab21 did not have an impact upon the early steps of endocytosis, though it did have an effect on the degradation pathway. These observations together with the colocalization of TbRab21 with TbRab28 and TbVps23 might suggest a role in late steps of endocytosis (Ali et al., 2014).

Orthologs of the ESCRT machinery, such as TbVps4, TbVps23, TbVps24, and TbVps28 were found in *T. brucei* (Leung et al., 2008; Silverman et al., 2013; Gilden et al., 2017; Umaer and Bangs, 2020). These ESCRT components as well as TbFab1 kinase and its product PI(3,5)P_2_, one of the ligands of TbVps24, localize in LE membranes of *T. brucei* (Leung et al., 2008; Silverman et al., 2013; Gilden et al., 2017; Umaer and Bangs, 2020). Despite the role of ESCRT in ILV formation and MVB development in mammalian cells, *T. brucei* lacks a well-defined MVB (Silverman et al., 2013). Another typical membrane-bound protein of LEs is TbRab7 (Engstler et al., 2004). TbRab7 depletion does not affect the endocytosis rate, but leads to complete cessation of delivery of the endocytosed cargo to the lysosome, a similar effect to that observed upon silencing of TbVps23 and TbVps4 (Silverman et al., 2011, 2013). Interestingly, while TbRab7 has no influence on biosynthetic trafficking of the lysosomal markers p67 and TbCathepsin L (TbCatL), the ESCRT components TbVps4, TbVps23 and TbVps24 do (Silverman et al., 2011, 2013; Umaer and Bangs, 2020). Another Rab, TbRab28, seems to be involved in transport to the lysosome. Depletion of the protein by RNAi resulted in reduced ConA transport to the lysosome in 80% of the cells (Lumb et al., 2011). LEs and lysosome localize proximal to the nucleus (Engstler et al., 2004). The transport from EEs to LEs and/or lysosome is performed by class II clathrin-coated vesicles (CCV II) found budding from endosomes (Grünfelder et al., 2003). CCVs II are 50–60 nm in diameter and are depleted in VSG and enriched in the fluid-phase markers ferritin and horseradish peroxidase (Grünfelder et al., 2003; Engstler et al., 2004). Interestingly, a detailed study of the kinetics of endocytosis in *T. brucei* showed that the fluid-phase marker dextran and biotinylated VSG (VSG_biotin_) were endocytosed at the same time and then were gradually segregated, reaching a maximum of spatial separation after approximately 1 min (Engstler et al., 2004). At steady state 37% of the intracellular VSG_biotin_ did not lie on the endocytic route of internalized dextran (Engstler et al., 2004). The authors suggested that the separation of VSG from the fluid-phase marker occurred concurrently with the biphasic filling of the RE (Engstler et al., 2004).

The RE is a compartment where cargo transported from EEs and LEs can be redirected to the plasma membrane (Grünfelder et al., 2003; Engstler et al., 2004; Overath and Engstler, 2004). This endosomal compartment is predominantly marked by TbRab11 and has, to a certain extent, an interface with EEs (Grünfelder et al., 2003; Chung et al., 2004; Engstler et al., 2004; Field et al., 2009). From the REs, the recycling cargo is destined for the FP via TbRab11-positive exocytic carriers. These disk-shaped carriers, with a diameter of ca. 154 nm and thickness of approximately 34 nm, were found close to both the FP and the cisternae-shaped endosomal compartment near the lysosome (Grünfelder et al., 2003; Engstler et al., 2004). Interestingly, electron microscopy revealed both VSG and horseradish peroxidase within the EXCs (Grünfelder et al., 2003; Engstler et al., 2004). Thus, TbRab11 has been proposed to be involved in both fluid-phase and receptor-mediated cargo recycling to the cell surface. Consistent with these results, TbRab11 RNAi depleted cells showed an approximately 80% reduction in recycling of transferrin (Hall et al., 2005). However, conflicting observations were reported in a further study that used the same knockdown strategy. In this study, TbRab11 depleted and control cells were treated with cycloheximide to flush nascent VSG from the exocytic pathway and free surface amino groups were blocked by acetylation at 4°C (Umaer et al., 2018). Subsequently, shifting to 37°C allowed the recycling of internal unblocked VSG, which is susceptible to surface biotinylation. Flow cytometry revealed an increase of about 10% in surface biotinylation 5 min after the temperature shift (Umaer et al., 2018). The authors considered this to be similar to the kinetics of exocytosis in control cells. This led to the proposition of the existence of a redundant mechanism to recycle VSG to the cell surface (Umaer et al., 2018). However, the endocytosis kinetics published by Engstler et al. (2004) reports that around 10% of total VSG (equivalent to the intracellular VSG pool) is recycled per minute. Consequently, the measurement after 5 min can mask an up to five times slower recycling kinetics and the possibility of a functional, but less efficient, recycling pathway without TbRab11. Another possibility could be related to the activity of residual TbRab11 following RNAi, which might still be contributing to VSG transport. It would be desirable to look at earlier timepoints (e.g., 1 min) to establish differences in kinetics of TbRab11 depleted and control cells. Therefore, the existence of a redundant mechanism in VSG recycling remains controversial.

A third class of clathrin-coated vesicles was observed close to the outermost *trans*-Golgi cisterna, which was proposed to be the TGN equivalent in *T. brucei* (Grünfelder et al., 2003). The TGN-homolog marker, TbGRIP70, was localized in the outer Golgi cisterna of the trypanosomatid *Leishmania mexicana* that had been genetically modified to express TbGRIP70, through immunoelectron microscopy (McConville et al., 2002). The same localization was observed in *T. brucei* through immunofluorescence assays using widefield microscopy (Sealey-Cardona et al., 2014). However, whether this third class of clathrin vesicles in trypanosomes is part of the biosynthetic route, as observed in HeLa cells infected with varicella-zoster virus (Alconada et al., 1996), or of the retrograde recycling route is not clear. Furthermore, homologs of the retromer complex subunits, associated with the retrograde transport in other organisms, were found in *T. brucei* (TbVps26, TbVps29, and TbVps35) as was one homolog of the SNX family interactor (TbVps5) (Koumandou et al., 2011). Immunofluorescence assays suggested a close association of the *T. brucei* retromer complex and the endocytic apparatus components (Koumandou et al., 2011). However, a structural characterization is not available to date. The depletion of TbRab28 led to a decrease in the expression of TbVps26 and the ESCRT components TbVps23 and TbVps28 suggesting that these components may have a functional connection (Lumb et al., 2011). Notably, the retrograde recycling mechanism is not associated with VSG (Engstler et al., 2004).

## Conclusion

In trypanosomes, most studies to identify adaptor/accessory proteins of the endocytic/exocytic machinery have employed homology searches based on genes found in mammals and yeast. Based on this, the trypanosome machinery has been deemed to be simpler than the machineries of the opisthokont models (Manna et al., 2014). However, genomic BLAST is limited to similarity searches, indicating not the complexity of the characteristics of different organisms but the conservation between them. Therefore, it is interesting and not entirely unexpected that studies using direct screening for trypanosome-specific proteins have led to the discovery of new components involved in distinct steps within the endocytic/exocytic machinery (Adung’a et al., 2013; Boehm et al., 2017).

Investigations into the maturation model and evidence of endosomal mobility are still missing from *T. brucei* cell biology. For example, our knowledge of the contribution of the cytoskeleton to vesicle budding and transport is limited. Consequently, it would be interesting to analyze transport mechanisms for directed vesicle motion in *T. brucei*. In addition, specific VSG sorting at different trafficking steps is likely to exist but remains to be studied.

The methods used for subcellular localization and morphological descriptions of the endocytic/exocytic apparatus are another important aspect to be considered. The compartment markers have usually been located via widefield fluorescence microscopy and characterized via RNAi knockdown. The use of electron microscopy has been restricted to the morphological analysis of the FP. However, considering the high dynamicity observed in endosomal tubules of living cells, analyses of fixed samples could give the impression of a fragmented morphology (Baetz and Goldenring, 2013; Goldenring, 2015). Furthermore, microscope resolution is an important limiting factor that could also lead to inaccuracies in morphological descriptions, as overlapping signals in epifluorescence microscopy can be resolved into subdomains inside an organelle when investigated by super-resolution microscopy (Baetz and Goldenring, 2014).

Therefore, our understanding of the divergent endomembrane system of *T. brucei* still lags behind that of the classical opisthokont systems. A greater focus on trypanosomes could contribute to a broader comprehension of cell biology. In fact, a more wholesome understanding of the biodiversity requires an in depth look at a range of diverged organisms. Detailing the dynamics of endocytic processes with nanoscale resolution using novel and emerging technologies, such as live correlative light and electron microscopy (live CLEM) (Fu et al., 2019) or expansion microscopy (Chen et al., 2015), will be the next step for all cell biology model systems, and this time there is no reason for the trypanosome model to lag behind.

## Author Contributions

AB and FL wrote the manuscript. AB designed the figures. ME and NJ provided conceptual input and contributed to writing. All authors read and approved the final manuscript.

## Conflict of Interest

The authors declare that the research was conducted in the absence of any commercial or financial relationships that could be construed as a potential conflict of interest.

## Publisher’s Note

All claims expressed in this article are solely those of the authors and do not necessarily represent those of their affiliated organizations, or those of the publisher, the editors and the reviewers. Any product that may be evaluated in this article, or claim that may be made by its manufacturer, is not guaranteed or endorsed by the publisher.
